# Does alcohol use have a causal effect on HIV incidence and disease progression? A review of the literature and a modeling strategy for quantifying the effect

**DOI:** 10.1186/s12963-017-0121-9

**Published:** 2017-02-10

**Authors:** Jürgen Rehm, Charlotte Probst, Kevin D. Shield, Paul A. Shuper

**Affiliations:** 1 Institute for Mental Health Policy Research, Centre for Addiction and Mental Health (CAMH), 33 Russell Street, Toronto, ON M5S 2S1 Canada; 2 Campbell Family Mental Health Research Institute, CAMH, 250 College Street, Toronto, ON M5T 1R8 Canada; 3 Institute of Medical Science (IMS), University of Toronto, Faculty of Medicine, Medical Sciences Building, 1 King’s College Circle, Room 2374, Toronto, ON M5S 1A8 Canada; 4 Institute for Clinical Psychology and Psychotherapy, Technische Universität Dresden, Chemnitzer Str. 46, 01187 Dresden, Germany; 5 Department of Psychiatry, University of Toronto, 250 College Street, 8th floor, Toronto, ON M5T 1R8 Canada; 6 Dalla Lana School of Public Health, University of Toronto, 155 College Street, 6th floor, Toronto, ON M5T 3 M7 Canada; 7 International Agency for Research on Cancer, 150 Cours Albert Thomas, 69372 Lyon, France

**Keywords:** Alcohol use, HIV, AIDS, Incidence, Course, Causality, Quantification of risk, South Africa

## Abstract

In the first part of this review, the nature of the associations between alcohol use and HIV/AIDS is discussed. Alcohol use has been found to be strongly associated with incidence and progression of HIV/AIDS, but the extent to which this association is causal has traditionally remained in question. Experiments where alcohol use has been manipulated as the independent variable have since helped establish a causal effect of alcohol use on the intention to engage in condomless sex. As the intention to engage in condomless sex is a surrogate measure of actual condom use behavior, which itself is linked to HIV incidence and re-infection, the causal chain has been corroborated. Moreover, there are biological pathways between alcohol use and the course of HIV/AIDS, only in part being mediated by adherence to antiretroviral medication. In the second part of the contribution, we provide suggestions on the quantification of the link between alcohol use and HIV incidence, using risk relations derived from experimental data. The biological links between alcohol use and course of HIV/AIDS are difficult to quantify given the current state of knowledge, except for an operationalization for the link via adherence to medication based on meta-analyses. The suggested quantifications are exemplified for South Africa.

**Electronic supplementary material:**

The online version of this article (doi:10.1186/s12963-017-0121-9) contains supplementary material, which is available to authorized users.

## Background

Alcohol use is a major risk factor for burden of disease and injury [1–4]. It has been shown to affect not only non-communicable chronic diseases [5, 6] and injuries [7, 8], but also communicable diseases [9, 10]. To date, two communicable diseases have been included into the Global Burden of Disease Comparative Risk Factor Assessment as being causally impacted by alcohol, starting with the 2010 study ([11]; see also the 2013 [4] and the 2015 [3] studies): tuberculosis [12, 13] and lower respiratory infections [14]. The Global Status Report on Alcohol and Health of the World Health Organization [1] included these two disease categories as well, but added the effect of alcohol use on antiretroviral medication adherence and its impact on AIDS mortality ([15]; based on a number of reviews [16–20]).

The status of alcohol use as a cause for HIV infection, and its effects separate from non-adherence, on the course of HIV/AIDS have been discussed in recent years [21–23] but were found to be non-conclusive at a meeting to discuss the causal role of alcohol use on HIV/AIDS organized by WHO and the South African Medical Research Council [24]. However, since this meeting took place, considerable new scientific evidence seems to support a causal role of alcohol. Furthermore, systematic reviews and meta-analyses are now available to allow quantification of the impact in a way in line with the quantification of other risks attributable to alcohol or other risk factors in the global Comparative Risk Assessments (for methodology see [25]). The present contribution first summarizes the evidence on the relationship between alcohol use and HIV/AIDS, mainly based on systematic reviews and meta-analyses. In the second part, a methodology to quantify this impact is introduced.

The establishment of causality is key in these deliberations. Causality is defined here based on Rothman [26–28] as a multiple components model, where all components need to be present to produce the effect. In the specific case for alcohol and HIV incidence, different such sets of components have been identified, which can be seen as causal pathways, where alcohol use plays a necessary role (see below). The case for causality seems convincing, because in addition to fulfilling the usual epidemiological and thus observational Bradford Hill criteria [29] such as association, temporality, and plausibility, we have experimental evidence for key parts of several pathways, including the pathway of alcohol use on decision-making.

## Review: Alcohol use and HIV/AIDS: association and causal considerations

Alcohol use was found to be associated with HIV in recent systematic reviews and meta-analyses [30–36]. Three different explanations have been brought forward to explain the association: a) the impact of alcohol use on decision-making, resulting in riskier sexual behaviors (reviews of [34, 37–43]); b) biological effects of alcohol use on HIV transmission and disease progression ([44] as overview; see also [16, 45–47]), including but not limited to effects of alcohol on treatment course and medication adherence [16–19, 48]; and c) that most or all of the effects of alcohol on HIV incidence and disease progression can be explained by third variables, especially the effect of risk-taking and other personality variables [49, 50].

To exclude the third explanation and corroborate the causality of the alcohol-HIV incidence via impacts on decision-making on safer sex practices, a number of experimental trials have been conducted, where alcohol use was experimentally manipulated as the main factor. Systematic reviews and meta-analysis of the results of these trials indicated a causal impact of alcohol use on decisions about unsafe sex practices, both when alcohol use was compared to placebo, or to intake of non-alcoholic beverages [42, 43]. It should be noted that the underlying experiments have been conducted in different populations, including people living with HIV (PLWH) (e.g., [51]).

The reservation must be made that for ethical and practical reasons, experimental studies, which are necessary to determine causality, can only use surrogate endpoints (i.e., intention for condomless sex) rather than condomless sex itself or HIV infection. However, the results of experimental studies corroborate the results of epidemiological cohort and cross-sectional studies with condomless sex [34, 37–39, 41, 52–55], sexually transmitted disease [56, 57], or HIV incidence [31] as endpoints. Moreover, there are meta-analyses that show a clear link between intentions for unsafe sex and resulting behavior [58, 59], as well as between condomless sexual practices and HIV seroconversion [60–62].

The above seems to be in contradiction to some event-level studies, which have not always found a negative impact of alcohol on unsafe sex [63, 64]. However, event-level studies have been often done in select non-probability samples, or via surveys without adequate control, so that the experimental evidence mentioned is clearly preferable to control for potential confounding [65].

With respect to biological impacts, there seems to be clear evidence that heavy drinking or alcohol use disorders are associated with viral load increases and/or CD4 count declines, general weakening of the immune system, and more negative outcomes of antiretroviral therapy (ART), partly mediated by treatment adherence and partly by the pharmacological interactions of alcohol with ART and other medications to treat co-morbidities (for the association regarding adherence see above; for the other associations see for heavy drinking: [46, 66]; for AUD: [16]; for mechanisms see [20, 46, 48, 67, 68]; for pharmacological interactions see [69, 70]). It should be noted, however, that delineation of causality in these biological pathways is difficult, as many factors interact, and even when causality has potentially been established, it is hard to quantify the causal contribution of alcohol to HIV/AIDS disease progression based on biological mechanisms [20, 66–68, 71].

## Quantification of the effect of alcohol use on HIV

### The effect of alcohol on decision-making

In a meta-analysis of experimental studies, [42] alcohol use associated with a blood alcohol concentration (BAC) level of 0.07 g/dl was shown to impact decision-making for condomless sex (based on averaging the 28 individual studies). The latter findings can be used for illustrative burden of disease quantifications. Participants with a BAC level of 0.07 g/dl were 1.54 (95% CI 1.31–1.78) times more likely to consider condomless sex compared to those who had not used alcohol (see also Additional file 1). For normal, non-experimental drinking situations, this BAC would correspond to an average of 49 g pure alcohol ingested for women and of 61 g for men. The grams of alcohol corresponding to a BAC of 0.07 g/dl were derived from standard tables based on sex-specific average body weight from the National Institute on Alcohol Abuse and Alcoholism (NIAAA) [72, 73].

In order to quantify the effect of alcohol use on HIV/AIDS at the population level, we selected people who consume on average at least at this level per day, corresponding to a small minority of the drinkers in each country. We provide the example of South Africa to illustrate these quantifications in a country with very high prevalence and burden of HIV/AIDS [74–76] and comparably high levels of heavy alcohol use among current drinkers [1] (see also Additional file 1).

There are several assumptions in this choice of operationalization. First, it assumes that sexually active people drink at least at the same level as the general population. Second, it assumes that people with HIV/AIDS continue to have sex and drink at the same level as the general population. And third, it is assumed that there are no interactions with other variables that could change the causal relation.

The first assumption seems to be without a problem; at least there is no empirical evidence for the contrary, and some specific evidence that sexual activity is associated with drinking in some groups. Regarding the second set of assumptions, alcohol consumption and HIV have been shown to be associated among cross-sectional samples in Africa [35]. This general association held true, despite the high stigma of alcohol use which may lead people with HIV or AIDS to withhold reporting their drinking status or drinking level, especially if they are seeking treatment for ART [77, 78]. Some of the results from Africa for people on ART showed less involvement with alcohol after treatment initiation, whereas other findings have demonstrated high alcohol consumption among ART patients [79, 80]. Regarding the assumption that people with HIV/AIDS continue to be sexually active, this has certainly been the case, even though evidence showed that their level of sexual activity and condomless sex may be reduced after testing positive in general, or after ART initiation ([81]; for papers specifically from South Africa: [82–84]). However, other evidence from South Africa suggested that alcohol use was associated with a high likelihood of condomless sex after a positive HIV test [85]. Overall, the main assumptions of the model about continuation of sexual activities and consumption of alcohol after seroconversion seem to be justified. The third assumption postulated lack of interactions. While we cannot control for all these interactions, such as use of vaginal microbicide gels [86] or socioeconomic status and related malnutrition [87], many of these seem to increase the impact of alcohol use on HIV infection.

### Illustrative quantification of the effect of alcohol use on HIV incidence in South Africa

Applying the above-derived drinking level cutoffs results in 29.6% (95% Uncertainty Interval (UI): 24.7%–32.3%) (15.6% women and 36.8% men; see all UIs in the Additional file 1) of adult (15 years of age and older) drinkers drinking above the cutoff; and 12.0% (95% UI: 9.3%–14.1%) (4.1% women and 20.7% men) of the total adult population. Combining this exposure, stratified by age, with the risk relations found in the most recent meta-analysis [42] resulted in the following estimates with respect to HIV incidence for South Africa in 2012: 18,200 incident cases (95% UI: 8,400–28,000) (7,000 among women, 11,200 among men) attributable to alcohol use; population attributable fraction 3.9% (95% UI: 1.8%–6.0%) (2.2% for women, 7.4% for men) (incidence data from [88]; for a detailed description of the methodology and all uncertainty intervals please see Additional file 1).

### Illustrative quantification of the effect of alcohol use on disease course and mortality including adherence to medication in South Africa

Combining the above-described sex- and age-specific exposure estimates with the risk relations reported in experimental studies yielded population attributable fractions of 4.5% (95% UI: 2.2%–6.8%) for HIV/AIDS mortality (2.3% for women and 6.8% for men) and 4.3% (95% UI: 2.1%–6.5%) (2.2% for women and 6.6% for men) for HIV/AIDS-related burden of disease, as quantified in disability-adjusted life years (DALYs) for 2012 in South Africa (for methodology see Additional file 1).

Combining this burden with the alcohol-attributable burden of disease caused by non-adherence to highly active ART (based on [15]), we estimate that in South Africa in 2012, alcohol use caused 12,200 (95% UI: 6,000–18,400) HIV- or AIDS-related deaths (2,900 deaths among women, 9,300 deaths among men); 634,800 (95% UI: 309,300–960,100) HIV/AIDS-related years of life lost due to premature mortality (YLL; 157,600 YLLs among women, 477,200 YLLs among men); 44,000 (95% UI: 21,300–66,700) HIV/AIDS-related years lived with disability (YLDs; 11,400 YLDs among women, 32,600 YLDs among men); and 678,800 (95% UI: 330,600–1,026,800) DALYs (169,100 DALYs among women, 509,700 DALYs among men). Uncertainty intervals for all estimates can be found in the Additional file 1.

## Discussion

The above estimates are likely to underestimate the alcohol-attributable HIV incidence and disease burden for a number of reasons. First, a dose–response relationship has not been quantified, and the estimate reported above was based on a step function with an increased risk for a BAC of 0.07 g/dl taken for all BACs above this threshold. However, a dose–response relationship where higher levels of BAC correspond with a higher risk to engage in/consider condomless sex can be expected. This relationship is hard to quantify, as BAC levels beyond 0.11 g/dl cannot be tested experimentally for ethical and practical reasons ([43]; for general dose–response relationships between alcohol and other behaviors such as drunk driving: [8]). It has also been shown that even below a BAC of 0.07 g/dl participants were roughly 50% more likely to report the intention of having condomless sex compared to those who did not drink alcohol [42]. However, the lower limits for the respective BAC were not quantified/reported.

Second, the above estimation is based on regular heavy use and thereby does not include single drinking occasions where a BAC above 0.07 g/dl is reached. This may have further contributed toward potential underestimation, as irregular heavy drinking occasions are a common drinking pattern in South Africa [89] and are generally more prevalent than regular heavy drinking (i.e., an average daily use above the threshold).

Finally, it should be noticed that the amount of alcohol needed to reach a specific BAC depends on body weight (e.g., [90]). The estimation of 49 g and 61 g of pure alcohol for women and men, respectively, is based on the average weight in the North American region. As the average weight in the African region is about 20 kg below the average weight in the North American Region [91], the amount of pure alcohol needed to reach a BAC of 0.07 g/dl can be considered as an overestimate for South Africa.

However, there may also be some attenuation of the risk relationship, as the link between intention and actual behavior is not perfect. This may be modeled in future analyses by including the strength of the association between intention and actual behavior from meta-analyses [58, 59].

Most important, however, is the fact that all the biological pathways of alcohol use (see above for discussion and references) are not considered in the current model. The above-derived population attributable fractions would also be markedly smaller than any fractions derived from the classic formulas [92, 93], combining the prevalence of drinking (around 40% in South Africa [1]) with relative risk for incident HIV (about 2 overall for all studies; [31]), which would result in a population-attributable fraction of 28%.

Overall, the above calculation presents a first approach to quantifying the impact of alcohol use on incidence of HIV and disease burden of HIV/AIDS, which goes beyond modeling only the effect on medication adherence. However, while this approach was able to estimate one additional pathway, there are other pathways such as the pharmacological effects on the immune system and the pharmacological interactions with medications, or the effect of alcohol use on re-infection [94], which remain to be quantified.

Moreover, different pathways may interact with each other. Even for the pathway modeled here, in the future continuous risk functions based on the continuous exposure distributions need to be created. Thus, while the current effort is one step to better modeling the impact of alcohol use on HIV incidence and burden of HIV/AIDS, more research on the above details is needed.

While the current paper has been confined to methodological aspects on the causality of the relationship between alcohol use and HIV/AIDS and on the quantification of identified pathways, the results of the review also have implications for health policy. Given the high impact of HIV/AIDS on the disease burden in South Africa and sub- Saharan Africa in general [74, 75, 95], the identification of alcohol as a causal factor for incidence opens new ways to reduce the disease burden of these conditions [94]. These include classic alcohol policy measures [96] as well as the integration of alcohol interventions into the treatment of HIV/AIDS [97].

## Conclusion

Alcohol use is strongly associated with the incidence and course of HIV/AIDS, and based on experimental data where alcohol use has been manipulated as the independent variable, a causal effect of alcohol use on the intention of condomless sex can be asserted. Condomless sex intentions can be seen as a surrogate measure of actual condom use behavior, which itself is linked to HIV incidence and re-infection. Moreover, there are biological links between alcohol use and worsening the course of HIV/AIDS, in part being mediated by adherence to medication.

To quantify the link between alcohol use and HIV incidence, we rely on risk relations based on experimental data. Other pathways from alcohol use to HIV/AIDS burden of disease cannot be quantified given the current state of knowledge, except for an operationalization for the link via adherence to medication based on meta-analyses. Overall, this approach to model alcohol-attributable incidence of HIV and burden of HIV/AIDS needs to be further developed in the future, especially with regard to accounting for dose–response relationships and binge drinking occasions.

## Additional file

Additional file 1: Supplemental methodological information. (DOCX 31 kb)

## Abbreviations

AIDS: Acquired Immunodeficiency Syndrome
ART: Antiretroviral therapy
AUD: Alcohol Use Disorders
BAC: Blood Alcohol Concentration
DALYs: Disability-adjusted life years
HIV: Human Immunodeficiency Virus
UI: Uncertainty interval
YLDs: Years lived with disability
YLLs: Years of life lost due to premature mortality
