# Supplementary material for: Does alcohol use have a causal effect on HIV incidence and disease progression? A review of the literature and a modeling strategy for quantifying the effect
Source: Popul Health Metr. 2017 Feb 10;15:4. doi: 10.1186/s12963-017-0121-9 (PMC5301358; doi:10.1186/s12963-017-0121-9)
Supplement: Additional file 1: — Supplemental methodological information. (DOCX 31 kb) [file 12963_2017_121_MOESM1_ESM.docx]

**Additional file: Supplemental methodological information**

To “Does alcohol use have a causal effect on HIV incidence and disease progression? A review of the literature and a modeling strategy for quantifying the effect”

## Modeling alcohol consumption in South Africa

Data on the prevalence of alcohol consumption, the per capita consumption of pure alcohol (estimated using recorded consumption (i.e., government statistics), and the unrecorded consumption (alcohol production not recorded by governments, such as illegal production and homebrew[[1](#_ENREF_1)]) for South Africa in 2012 were obtained from the Global Information System on Alcohol and Health (GISAH) ([[2](#_ENREF_2)]; for a description of the process to obtain data see [[3](#_ENREF_3),[4](#_ENREF_4)]). The amount of alcohol consumed among current drinkers was estimated using 90% of the number reported in the data as reported by the per capita alcohol consumption statistic [[5](#_ENREF_5)]. This discounting accounts for alcohol which is not consumed, as well as for the underestimation of alcohol consumption that is likely present in medical observational studies [[6](#_ENREF_6)]. The distribution of alcohol consumption among current drinkers (those who have consumed alcohol but have not done so in the past year) was modeled based on the methods of Rehm and colleagues [[7](#_ENREF_7),[8](#_ENREF_8)]. This method assumes that alcohol consumption among current drinkers is adequately modeled using a gamma distribution in all cases (see population survey review by [[8](#_ENREF_8)]) and that there is a strong relationship between the mean alcohol consumption among current drinkers and the standard deviation, such that the standard deviation of the alcohol consumption distribution can be predicted using the mean alcohol consumption among current drinkers.

## Population attributable fraction for new HIV infections.

The fraction of incident cases of HIV and the fraction of deaths, years of life lost and years lived with disability were estimated using a population attributable fraction (PAF) methodology [[9](#_ENREF_9)]. This methodology combines data on alcohol consumption in a country and the relative risks (RRs) and uses the theoretical minimum risk for alcohol consumption of lifetime abstention (those who have not consumed at least one standard drink of alcohol in their lifetime). The PAF was estimated using Formula 1:

(Formula 1)

$$PAF=\frac{P_{Abs}+\int_{>0}^{150} P_{CD}\left( x \right){RR}_{CD}\left( x \right)dx-1}{P_{Abs}+\int_{>0}^{150} P_{CD}\left( x \right){RR}_{CD}\left( x \right)dx}$$

Within Formula 1, P_abs_ represents the prevalence of abstention (both lifetime and current abstention, the latter being defined as having had no drink in the past year), P_CD_(x) represents the prevalence of current drinkers who consume a given amount of alcohol (denoted as x). Furthermore, RR_CD_(x) represents the RR of becoming infected with HIV for current drinkers (who consume a given amount of alcohol x) as compared to lifetime abstainers. In this estimation of the alcohol PAF of new HIV infections, the RR for abstainers is assumed to be equal to 1 (i.e., the risk of new HIV infection among abstainers).

The relative risk (in this case an odds ratio) used in the present study was calculated from the effect size d reported by Scott-Sheldon and colleagues [[10](#_ENREF_10)] using Formula 2 ([[11](#_ENREF_11)], chapter 7):

(Formula 2)

$$LogOddsRatio=d*\frac{\pi}{\sqrt{3}}$$

The resulting OR was 1.54 (95% CI 1.31–1.78).

## Alcohol population attributable fraction for non-adherence to highly active antiretroviral therapy

Estimates of excess HIV/AIDS mortality attributable to non-adherence to highly active antiretroviral therapy (HAART) due to the consumption of alcohol are outlined elsewhere [[12](#_ENREF_12)]. These estimates combine data on HIV/AIDS mortality, the prevalence of people who require and are receiving HAART (obtained from the Report on the Global AIDS Epidemic), the mortality risk for those individuals who do and do not receive HAART [[13](#_ENREF_13)], the mortality risk for those individuals who adhere to HAART and for those who do not [[14](#_ENREF_14)], and the hazard of not adhering to HAART for current drinkers as compared to lifetime abstainers [[15](#_ENREF_15)].

## Estimation of uncertainty intervals

The estimation of the 95% uncertainty intervals (UIs) was obtained from a Monte Carlo simulation (see [[16](#_ENREF_16)] for more details). This simulation estimated the UIs by drawing 40,000 samples of the lowest level parameters from their respective random error distributions, and then using these estimates to formulate 40,000 PAFs. The variance of these PAFs was then used to estimate the error distribution of the PAFs and construct 95% UIs.

## Uncertainty intervals for estimates presented

**Table A1.** Prevalence of heavy drinkers among adults (15 years of age and older) in South Africa, 2012

| **Prevalence** | | **Point estimate** | **Lower estimate** | **Upper estimate** |
| --- | --- | --- | --- | --- |
| **Among current drinkers** | |  |  |  |
|  | Total | 29.6% | 24.7% | 32.3% |
|  | Men (>= 61 grams per day) | 36.8% | 33.6% | 38.7% |
|  | Women (>= 49 grams per day) | 15.6% | 6.3% | 20.4% |
| **Among the total population** | |  |  |  |
|  | Total | 12.0% | 9.3% | 14.1% |
|  | Men (>= 61 grams per day) | 20.7% | 17.9% | 23.0% |
|  | Women (>= 49 grams per day) | 4.1% | 1.5% | 5.9% |

**Table A2.** Alcohol-attributable fractions caused by HIV infections contracted during condomless sex attributable to alcohol consumption in South Africa, 2012

| **Prevalence** | | **Point estimate** | **Lower estimate** |  | **Upper estimate** |
| --- | --- | --- | --- | --- | --- |
| Incidence | |  |  |  |  |
|  | Total | 3.9% | 1.8% |  | 6.0% |
|  | Men | 7.4% | 4.2% |  | 10.6% |
|  | Women | 2.2% | 0.7% |  | 3.8% |
| Deaths | |  |  |  |  |
|  | Total | 4.5% | 2.2% |  | 6.8% |
|  | Men | 6.8% | 3.8% |  | 9.8% |
|  | Women | 2.3% | 0.6% |  | 3.9% |
| Years of life lost | |  |  |  |  |
|  | Total | 4.3% | 2.1% |  | 6.5% |
|  | Men | 6.6% | 3.7% |  | 9.4% |
|  | Women | 2.2% | 0.7% |  | 3.8% |
| Years lived with disability | |  |  |  |  |
|  | Total | 4.4% | 2.1% |  | 6.7% |
|  | Men | 6.8% | 3.8% |  | 9.8% |
|  | Women | 2.3% | 0.7% |  | 4.0% |
| Disability-adjusted life years | |  |  |  |  |
|  | Total | 4.3% | 2.1% |  | 6.5% |
|  | Men | 6.6% | 3.7% |  | 9.5% |
|  | Women | 2.2% | 0.7% |  | 3.8% |

**Table A3.** Incident cases of HIV attributable to alcohol consumption in 2012 in South Africa and the burden of HIV attributable to alcohol consumption in 2012 in South Africa.

| **Prevalence** | | **Point estimate** | **Lower estimate** | **Upper estimate** |
| --- | --- | --- | --- | --- |
| Incidence | |  |  |  |
|  | Total | 18,200 | 8,400 | 28,000 |
|  | Men | 11,200 | 6,300 | 16,000 |
|  | Women | 7,000 | 2,100 | 12,000 |
| Deaths | |  |  |  |
|  | Total | 12,200 | 6,000 | 18,400 |
|  | Men | 9,300 | 5,100 | 13,600 |
|  | Women | 2,900 | 900 | 4,800 |
| Years of life lost | |  |  |  |
|  | Total | 634,800 | 309,300 | 960,100 |
|  | Men | 477,200 | 259,200 | 695,200 |
|  | Women | 157,600 | 50,200 | 264,800 |
| Years lived with disability | |  |  |  |
|  | Total | 44,000 | 21,300 | 66,700 |
|  | Men | 32,600 | 17,700 | 47,400 |
|  | Women | 11,400 | 3,600 | 19,300 |
| Disability-adjusted life years | |  |  |  |
|  | Total | 678,800 | 330,600 | 1,026,800 |
|  | Men | 509,700 | 276,800 | 742,700 |
|  | Women | 169,100 | 53,800 | 284,100 |

## References

1. Rehm J, Kailasapillai S, Larsen E, Rehm MX, Samokhvalov AV, et al. (2014) A systematic review of the epidemiology of unrecorded alcohol consumption and the chemical composition of unrecorded alcohol. *Addiction* 109: 880-893.

2. World Health Organization (2015) *Global Information System on Alcohol and Health*. Geneva, Switzerland:World Health Organization Report.

3. Poznyak V, Fleischmann A, Rekve D, Rylett M, Rehm J, et al. (2013) The World Health Organization’s Global Monitoring System on Alcohol and Health. *Alcohol Res* 35: 244-249.

4. Rehm J, Poznyak V (2015) On monitoring unrecorded alcohol consumption. *Alkoholizm i Narkomania (Alcoholism and Drug Addiction)* 28: 79-89.

5. Rehm J, Klotsche J, Patra J (2007) Comparative quantification of alcohol exposure as risk factor for global burden of disease. *Int J Methods Psychiatr Res* 16: 66-76.

6. Gmel G, Rehm J (2004) Measuring alcohol consumption. *Contemp Drug Probl* 31: 467-540.

7. Rehm J, Kehoe T, Gmel G, Stinson F, Grant B, et al. (2010) Statistical modeling of volume of alcohol exposure for epidemiological studies of population health: the example of the US. *Popul Health Metr* 8: 3.

8. Kehoe T, Gmel G, Jr., Shield K, Gmel G, Sr., Rehm J (2012) Determining the best population-level alcohol consumption model and its impact on estimates of alcohol-attributable harms. *Popul Health Metr* 10: 6.

9. Ezzati M, Vander Hoorn S, Rodgers A, Lopez AD, Mathers CD (2003) Estimates of global and regional potential health gains from reducing multiple major risk factors. *The Lancet* 362: 271-280.

10. Scott-Sheldon LA, Carey KB, Cunningham K, Johnson BT, Carey MP (2015) Alcohol Use Predicts Sexual Decision-Making: A Systematic Review and Meta-Analysis of the Experimental Literature. *AIDS Behav Suppl* 1: S19-S39.

11. Borentstein M, Hedges LV, Higgins JPT, Rothstein HR (2009) *Introduction to meta-analysis*. Chichester, UK: John Wiley & Sons Ltd.

12. Gmel G, Shield K, Rehm J (2011) Developing a methodology to derive alcohol-attributable fractions for HIV/AIDS mortality based on alcohol's impact on adherence to antiretroviral medication. *Popul Health Metr* 9: 5.

13. Murphy EL, Collier AC, Kalish LA, Assmann SF, Para MF, et al. (2001) Highly Active Antiretroviral Therapy decreases mortality and morbidity in patients with advanced HIV disease. *Ann Intern Med* 135: 17-26.

14. Lima VD, Harrigan R, Murray M, Moore DM, Wood E, et al. (2008) Differential impact of adherence on long-term treatment response among naïve HIV-infected individuals. *AIDS* 22: 2371-2380.

15. Hendershot CS, Stoner SA, Pantalone DW, Simoni JM (2009) Alcohol use and antiretroviral adherence: review and meta-analysis. *J Acquir Immune Defic Syndr* 52: 180-202.

16. Gmel GJ, Shield KD, Frick H, Kehoe T, Gmel G, et al. (2011) Estimating uncertainty of alcohol-attributable fractions for infectious and chronic diseases. *BMC Med Res* *Methodol* 11: 48.
